# Women’s and health providers’ perceptions of companionship during labor and childbirth: a formative study for the implementation of WHO companionship model in Burkina Faso

**DOI:** 10.1186/s12978-023-01597-w

**Published:** 2023-03-21

**Authors:** Fadima Yaya Bocoum, Charles Paulin Kabore, Saran Barro, Roger Zerbo, Simon Tiendrebeogo, Claudia Hanson, Alexandre Dumont, Ana Pilar Betran, Meghan A. Bohren

**Affiliations:** 1 Institut de Recherche en Sciences de la Santé, Ouagadougou, Burkina Faso; 2 African Population Health Research Center, Dakar, Senegal; 3 INSS-CNRST/LARISS et CEFORGRIS-UJKZ/IRL-3189 “Environnement Santé et Sociétés”, Ouagadougou, Burkina Faso; 4 Department of Global Public Health, Karolinska Institutet, 17177 Stockholm, Sweden; 5 Department of Disease Control, London School of Hygiene and Tropical Medicine, London, UK; 6 CEPED, Institute for Research on Sustainable Development, IRD-Université de Paris, ERL INSERM SAGESUD, Paris, France; 7 UNDP/UNFPA/UNICEF/World Bank Special Program of Research, Development and Research Training in Human Reproduction (HRP), Department of Sexual and Reproductive Health and Research, World Health Organization, Geneva, Switzerland; 8 Gender and Women’s Health Unit, Centre for Health Equity, School of Population and Global Health, University of Melbourne, Carlton, VIC Australia

**Keywords:** Companionship, Policy, Barriers, Enablers, Burkina Faso

## Abstract

**Introduction:**

A key component of achieving respectful maternal and newborn care is labor companionship. Despite important health benefits for the woman and baby, there are critical gaps in implementing labor companionship for all women globally. The paper aims to present the perceptions and experiences of pregnant women, postpartum women, and health care providers regarding companionship during labor and childbirth, and to identify barriers and facilitating factors to the implementation of labor companionship in Burkina Faso.

**Methods:**

This is a formative study to inform the “Appropriate use of cesarean section through QUALIty DECision-making by women and providers” (QUALI-DEC) study, to design, adapt and implement a strategy to optimize the use of the cesarean section, including labor companionship. We use in-depth interviews (women, potential companions, and health workers) and health facility readiness assessments in eight hospitals across Burkina Faso. We use a thematic analysis approach for interviews, and narrative summaries to describe facility readiness assessment.

**Results:**

In all, 77 qualitative interviews and eight readiness assessments are included in this analysis. The findings showed that all participants acknowledged an existing traditional companionship model, which allowed companions to support women only in the hospital waiting room and post-natal room. Despite recognizing clear benefits, participants were not familiar with companionship during labor and childbirth in the hospital as recommended by WHO. Key barriers to implementing companionship throughout labor and birth include limited space in labor and delivery wards, no private rooms for women, hospital rules preventing companionship, and social norms preventing the choice of a companion by the woman.

**Conclusion:**

Labor companionship was considered highly acceptable in Burkina Faso, but more work is needed to adapt to the hospital environment. Revisions to hospital policies to allow companions during labor and childbirth are needed as well as changes to provide private space for women. Training potential companions about their roles and encouraging women’s rights to choose their companions may help to facilitate effective implementation.

## Plain Language Summary

A labor companion is a person who supports a woman throughout labor and birth, typically a woman’s spouse/partner, family member, or friend. We were interested to explore if a labor companion throughout labor and birth was acceptable to women and health workers, and feasible to start doing for women in Burkina Faso. To achieve this objective, we interviewed 77 women, companions, and health workers about their beliefs and opinions about labor companions, and what might help or be a challenge to having companions. We also visited eight hospitals to understand what the labor ward looks like, and any policies about companions. We found that hospitals were allowing a ‘traditional model’ of companionship, allowing companions to accompany women only in the waiting and postnatal rooms (not during labor or birth). Both women and health workers believed that companionship during labor and birth would be helpful and desired, which might help to make it a reality for all women. We also identified some challenges, due to crowded labor rooms with limited privacy, and hospital rules that did not allow companionship. We found that sometimes a woman’s family would choose the companion, instead of the woman herself, which may mean the woman does not want this person present. We plan to use these findings to help us to develop a program that allows any woman to have a companion present during labor and birth, in a way that ensures that she chooses the companion, and the companion can support her well.

## Introduction

Globally, improving the quality of care during childbirth has become a major concern, as births occurring in health facilities are increasing throughout many low- and middle-income countries (LMICs) [1]. The World Health Organization (WHO) defines the quality of care as both the provision of technically competent care and the enhancement of women’s experiences of care [2]. It acknowledges the importance of women and families receiving effective communication, respect, and emotional support as critical on the pathway to improving maternal health. A key component of achieving respectful maternal and newborn care is labor companionship, which is recommended by WHO [3]. WHO defines a labor companion as a person of the woman’s choice (such as a spouse/partner, female friend or relative, community member, or doula) who provides support continuously throughout labor and childbirth [1]. Labor companionship has important health benefits including reductions in cesarean section (C-section), shorter duration of labor, and better childbirth experiences [4]. Labor companions can help to improve communication and understanding between women, families and health workers, can facilitate non-pharmacological pain relief such as massage, mobility and changing positions, and can provide important emotional support, praise, and reassurance to the woman [5]. These benefits to women’s health and well-being can ultimately improve birth experiences, better engage family or other social support for women during labor, and build women’s confidence in their abilities to give birth [4, 5]. Despite the recognized benefits of labor companionship, implementation—especially in LMICs—remains suboptimal [6]. Known barriers to the implementation of labor companionship include limited training for women, families, and health workers on the benefits of companionship, restrictive policies at the health facility level, and a lack of space or privacy in health facilities [5].

### The QUALI-DEC project

In response to the significant increase in the rate of C-sections worldwide, WHO stated in 2015 that “Every effort should be made to provide C-sections to women in need, rather than striving to achieve a specific rate” [7]. High rates of C-sections can expose women who do not need them and their babies to unnecessary risk and serious complications, especially when performed in a limited-resource environment. Non-clinical interventions have been shown to safely reduce C-section rates, predominantly in high-income settings [8]. These interventions typically either target women and families through improved antenatal education and support, or target clinicians who are involved in C-section decision-making (doctors and midwives). To respond to rising rates of C-sections, a consortium of researchers initiated the ‘QUALIty DECision-making by women and providers for appropriate use of C-section’ (QUALI-DEC) project, with interventions to reduce unnecessary C-section [9]. The QUALI-DEC strategy is designed to combine four key interventions: (1) opinion leaders (influential obstetricians in each hospital) to implement evidence-based clinical guidelines; (2) audit and feedback using the Robson classification, (3) a decision-analysis tool to help women make an informed decision on mode of birth; and (4) implementation of WHO recommendations on labor companionship. The research project is implemented in four countries: Burkina Faso, Argentina, Viet Nam, and Thailand.

Ahead of the implementation of the QUALI-DEC trial, we conducted formative research to better understand the implementation and social context in Burkina Faso. To date, there is very limited research in Burkina Faso about women’s experiences of maternity care, including labor companionship [10]. This study aims to present the perceptions and experiences of pregnant women, postpartum women, and health care providers regarding labor companionship, and to identify barriers and facilitating factors to the implementation of the WHO companionship model in Burkina Faso.

## Methods

### Study context

Located in the heart of West Africa, Burkina Faso is a landlocked country. The health system is pyramidal and organized into three levels of care to provide primary, secondary, and tertiary care. The first level corresponds to the health district, which comprises two levels: (1) the primary health care center (PHCC) called “Centre de Santé et de Promotion Sociale” (CSPS) and (2) the Medical Centre without Surgical Unit (CMU). The secondary level of care is the Medical Centre with Surgical Unit (MCS), which is the reference center for the district’s health facilities. The third level is represented by the regional hospital and University hospital, which is the reference for the MCS. The university hospital is the highest level of reference. The maternal mortality ratio in Burkina Faso was estimated at 320 per 100,000 live births in 2018 [11]. In 2020, the rate of skilled attendance at birth was 77.2% and the national C-section rate, was 2.6% [12], however large inequalities exist [13, 14].

In Burkina Faso, women were traditionally assisted during labor and birth by *poko rogsa* (traditional birth attendants in the Mooré language), and other women in the community. At present, even if women are assisted by health workers at a health care facility, they are typically accompanied to the health care facility by companions, mainly women. National guidelines in Burkina Faso state that as part of antenatal care provision in first-level health facilities, pregnant women are to develop a birth plan in collaboration with healthcare providers [15]. The birth plan should include the identification of the labor companion, who should be aware of her/his role by a healthcare provider during antenatal care [15]. The national guidelines also provide information on how staff should work with companions and on the role of companions, typically to act as a link between the family and health workers, to support women, to bring food or drink to the woman, to bring samples and test results, and to purchase medicines [15]. The health providers are responsible for explaining these roles to the companions. According to the national guidelines, the companion should always seek to be at the side of the woman in labor and may also perform simple tasks such as: helping the woman to breathe and relax, massaging her back, caressing her belly, and giving her something to drink. The companion should seek a health professional if the woman shows danger signs such as bleeding, seizures, severe pain, or other problems. The companion should not encourage the woman to push, give advice different from that given by the health worker, prevent the woman from leaving her bed if she wants to move, or administer herbs or traditional remedies.

Despite national guidance on labor companionship, there are challenges with implementation. For example, the woman herself may not be able to choose her companion; rather, the companion may be chosen by the family, typically the mother-in-law, sister, or sister-in-law. The labor companion may only be allowed to be admitted to the labor or delivery room under certain conditions or certain time periods, typically for the first stage of labor only, then in the immediate postpartum period (first or second hour) and postnatal ward. The labor companion is typically not allowed to be present to support the woman during the second stage of labor or birth (either vaginal or cesarean birth).

### Study design

The protocol for this research was adapted from the generic formative research protocol proposed by WHO, which was designed to guide the design and implementation of interventions to reduce unnecessary C-sections [16]. The formative research consists of three main research activities: (1) document review; (2) facility readiness assessment; and (3) qualitative study. This paper presents findings from the facility readiness assessment and the qualitative study in Burkina Faso.

The facility readiness assessment consisted of describing and assessing the service delivery context. Non-participant observation of the labor and delivery ward and medical records were conducted. Where possible, photos of equipment were taken. A form was developed for this purpose and filled for each facility. The qualitative study consisted of in-depth interviews with women and their relatives, healthcare providers and administrators.

### Study sites

The QUALI-DEC study in Burkina Faso includes eight hospitals distributed among 6 regions (Table 1). The hospitals were purposively selected according to the programmatic activities and priorities of the country and geographical representation of the regions and its hospitals. The readiness assessment was conducted in all eight QUALI-DEC hospitals, and four hospitals (hospital 1, 5, 6, 7 in Table 1) were purposively selected for the primary qualitative component.

**Table 1:** Number of births and caesarean section rate per health facility in 2020

Facility	Region	Type of hospital	Number of births in 2020^a^	Cesarean section rate in 2020^a^ (%)
Hospital 1	Centre	Public teaching hospital	5324	44
Hospital 2	Hauts-Bassins	Public teaching hospital	4639	29
Hospital 3	Centre Ouest	Public hospital	3850	33
Hospital 4	Nord	Public teaching hospital	3086	25
Hospital 5	Centre Est	Public hospital	2207	33
Hospital 6	Centre Nord	Public hospital	3042	32
Hospital 7	Hauts Bassins	Public hospital	NA	16
Hospital 8	Centre	Public hospital	NA	21

NA: data for these hospitals are for the health district

^a^Data are from the yearbook 2020 of the ministry of health

### Participants and recruitment

There were five groups of participants in this study: pregnant women, postpartum women, companions, potential companions, and health care providers and administrators. We prespecified the target sample size for each type of participant, as informational power suggests that high quality data collection, variability of participant experience and background, and sample sufficiency are critical factors to determining appropriate sample sizes in qualitative research [17].

Pregnant and postpartum women aged 18 to 49 years who received antenatal and postpartum care in the study health facilities were invited to participate in in-depth interviews. Postpartum women who gave birth in the study health facilities in the previous 24 h were also invited to participate. The health workers facilitated contact with women during their visit or stay to the health facility. The health workers introduced the research team who invited eligible women to participate. For both pregnant and postpartum women, the research team ensured that a diverse group of women were included with a mix of residence (urban/rural), parity, age, ethnicity, and religion. The research team also aimed to include women with diverse obstetric histories (e.g., with and without previous C-section), and, for postpartum women, those who underwent vaginal and cesarean birth in the index birth. The sample target per facility was 3–6 pregnant and 2–4 postpartum women).

Pregnant and postpartum women who participated in the research identified potential companions (before or after birth). Potential companions before birth were people whom the pregnant woman would like to have as companion during the labor and birth. Potential companions after birth, were people who either supported the postpartum woman during her labor and birth, or whom the woman would have liked to support her. A potential companion was interviewed if he or she was present with the woman at the antenatal care visit, or during the hospital stay in postpartum period, prior to the woman’s discharge. Some interviews were conducted with potential companion outside the facility. The sample target per facility was 2–4 potential companions before birth and 1–2 after birth).

We also included purposively selected healthcare providers, including medical doctors (obstetricians, residents, interns), nurses and midwives working in the antenatal and delivery ward of the study facilities with a view of diversity in terms of position, gender, and years of experience. Healthcare administrators including managers of the maternity ward or health facility (e.g., medical/clinical director, head of obstetrics, midwife-in-charge) were also invited to participate in in-depth interviews. Providers were contacted by the research team at their workplace and asked to choose a convenient place for interview. The sample target per facility was 1–2 midwives, 1–2 doctors, 1–2 administrators). Table 2 shows the final sample included in the study.

**Table 2:** Number of participants per profile and facility included in the study

	Administrator	Health providers	Pregnant women	Companion	Postpartum women	Total
Hospital 1	2	6	6	3	4	21
Hospital 5	2	4	5	3	4	18
Hospital 6	2	2	5	4	4	17
Hospital 7	2	5	6	4	4	21
Total	8	17	22	14	16	77

### Data collection and tools

Data were collected by four female social scientists who had experience in qualitative research. They received a 3-day training prior to data collection. Data were collected in February 2020. Each potential participant was provided with information about the study and invited to participate by the data collector. In-depth interviews (IDIs) took between 30 and 90 min to complete, and took place in a comfortable and convenient location, such as a private room of the hospital, which was free from interruptions. Sociodemographic information was collected for each participant. Pregnant and postpartum women received soaps as compensation for the time devoted to the interview.

The semi-structured discussion guide for each six participant groups was developed based on previous work [16]. During the interviews, the WHO companionship model was explained to women and healthcare providers including the provision of support continuously throughout labor, birth, and postnatally, and the choice of the companion by the woman. The interviews were conducted in French and local languages including Mooré and Dioula. All interviews were audio recorded then transcribed in French by experienced transcribers who speak and understand local languages and French. Transcripts were anonymized during the transcription process. The study was performed under the supervision of the social scientist to ensure quality control.

The readiness assessment was carried out in the eight hospitals by a medical doctor who was not employed by any of the selected hospitals. Data collection occurred from 17 February to 7 March 2020. Prior to conducting the data collection, all members of the maternity care unit at each health facility were briefed on the purpose of the activity, what the readiness assessment entailed, and how they could assist during the data collection.

A semi-structured form was used to observe service delivery context in each facility settings. The key topics covered by the readiness assessment were: (1) Inventory of physical space and resources; (2) Health workforce and model of care; (3) Protocols and guidelines for managing clinical care during labor and childbirth; (4) Continuous education and quality improvement; (5) Assessment of facility medical records and data management systems; and (6) Labor companionship in practice [16]. The topics related to facilitators and barriers to labor companionship, such as the physical environment in labor room and postpartum room, were integrated in this paper.

### Data management and analysis

All transcripts were imported into NVIVO 11 software (QSR international, Doncaster, Australia). The data analysis was conducted in French, with quotes for this paper translated to English at the time of writing. A bilingual locally based social scientist member of the research team was responsible for translation of quotes from French to English to minimize the interpretation lost. A thematic analysis approach was used [18]. This analysis is appropriate to understand a set of experiences, thoughts, or behaviors within the data [18]. The analysis was performed according to the following steps: (a) organizing the data; (b) generating categories, themes, patterns; (c) testing emergent hypotheses; (d) searching for alternative explanations and deviant cases. We used a combined inductive and deductive approach. The deductive approach was used by generating a codebook based on the objectives and themes from the guides. Then, codes from the codebook were applied to the data. An inductive approach was used for initial coding to identify other themes that emerged naturally from the data. Responses to the topics in the readiness assessment were combined with the findings from the qualitative research to identify barriers and to develop considerations for implementation of the WHO companionship model.

Key themes emerging from the qualitative analysis were used to inform the analysis of data from the readiness assessment. The readiness assessment consisted primarily of short answer and open-ended questions, which were analyzed descriptively to provide more information about the hospital context and environment. Together, the analyses of qualitative interviews and readiness assessment provide a holistic picture of the care environment and peoples’ perceptions and experiences in receiving and providing care.

### Ethics considerations

The study was approved by the institutional health research ethics committee of the Institut de Recherche en Sciences de la Santé (IRSS) (A021-2019) in Burkina Faso. It was also approved by the Comité Consultatif Ethique pour la Recherche en Partenariat at IRD in France (notice of 6 April 2020), and the Research Project Review Panel (RP2) of the UNDP/UNFPA/UNICEF/WHO/World Bank Special Programme of Research, Development and Research Training in Human Reproduction (HRP) at the Department of Sexual and Reproductive Health and Research of WHO. Finally, the WHO Research Ethics Review Committee (ERC), Geneva, Switzerland approved the protocol.

Before starting the interview, eligible participant received information (objectives, procedures, benefits, duration of the interview) about the study in their preferred language. Women who consented to participate in the study were requested to sign the informed consent form. Women with low levels of literacy were able to sign with a thumbprint. Women participating in the study received soaps. Other participants (such as providers and stakeholders) did not receive incentives for participation. For the readiness assessment, women and providers were similarly asked to provide informed consent for non-participant observations of care provision.

All data including data collection forms, observation grids and transcripts were pseudonymized using unique participant numbers, with identifier codes stored in a password-protected folder accessible only to designated study team members.

## Results

Table 3 presents socio-demographic characteristic of women and companions. A total of 22 pregnant women were interviewed, almost all were married, ranged in age from 20 to 40 years old, and had an average age of current pregnancy of 6 months. Most were housewives (31.8%), students (22.7%), or sellers (13.6%). A total of 16 postpartum women were interviewed, almost all were married, ranged in age from 19 to 40 years, and most were housewives or farmers (50%), or sellers (18.8%), or students (18.8%). Half gave birth by C-section and half by vaginal birth. A total of 14 companions were interviewed, and most were women [11], and ranged in age from 27 to 61 years. There were two widows and 12 married companions and were from different socio-professional categories such as housewives (28.7%), and sellers (28.6%). Most of them were relatives [10] such as mother, husband, sister, aunt, or in-law.

**Table 3:** Sociodemographic characteristic of pregnant and postpartum women and companions

	Pregnant woman (n = 22)	Postpartum woman (n = 16)	Companion (n = 14)
Marital status			
Married	20	15	12
Other	2	1	2
Age (years)			
Mean	29	26.5	44
Category age (lowest-highest)	20–40	19–40	27–61
Occupation			
Housewife	7	6	4
Student	4	3	0
Seller	3	3	4
Other	8	4	6

Eight administrators and 17 health providers were interviewed: nine gynecologists, one general practitioner, nine midwifes and six nurses. There were 15 men and 10 women ranging in age from 32 to 52 years. In terms of professional experience the lowest was 2 years and the highest 22 years.

### Contextual environment from the readiness assessment

The eight hospitals had no specific private rooms for women in the first (latent) phase of labor. Instead, women in the latent phase of labor wait in the corridors or the halls of the maternity ward with their companions. There was limited private space for women in the delivery room. In five hospitals, there was a wall or curtain for privacy, but the delivery rooms were typically occupied by more women than available beds.

Maternity wards adhered to hospital visiting hours. In the post-natal/post-operative department, visiting hours were: 6–6:45 am, 12–2:30 pm, and 5–9 pm on weekdays. On weekends and public holidays, the visiting hours were from 10 am to 9 pm. Visits are not allowed in the delivery room in any of the hospitals. However, health workers sometimes provided an authorization note to allow free mobility to one companion of woman in the maternity ward and delivery room when necessary. There were no specific accommodation or comfort facilities for family members and companions of women, such as a chair, additional bed space, or toilets. Most of the time, companions slept in the same room with the postpartum women. If the postnatal ward was not full (i.e., some beds were not occupied by postpartum women), then companions could sleep on the available beds or lie down on a mat next to the woman’s bed.

### Findings from the in-depth interviews

In this section, the qualitative findings were related to the (1) knowledge of labor companionship, (2) the perceived benefits and potential risks of labor companionship, (3) the current and expected role of companions, and (4) enablers and barriers to the implementation of labor companionship.

#### Knowledge about companionship

While health workers were mostly not aware of the WHO definitions of labor companions, they were aware of the local system of companionship, as stated by this administrator:

“*Most of the time there is always a companion next to her when she gives birth*” (Midwife, administrator, hospital 1).

Most of the pregnant and postpartum women interviewed positively valued labor companionship, but were not aware of the type of companionship promoted by WHO:

“*I think that this support is welcome, because it has been said that if a woman is taken care of psychologically, morally, physically, she will have all the support and the labor will go well*” (Postpartum woman with C-section, hospital 7).

Women expressed that they would like to have a labor companion who could stay with them throughout labor and birth and act as a trusted companion. But many of them wondered where or how to find this trusted person as stated by this woman:

“*I pray to have one like this, but I don't have one at the moment*” (Pregnant woman with no history of C-section, hospital 1)

#### Perceived benefits and potential risks of companionship

All respondents recognized the advantages of companionship and described two main benefits for women: (1) psychological and moral support during labor and birth, and (2) practical support. First, companions provided psychological and moral support by reassuring the woman and helping to support her through the pain. For example, one woman described:

“*If you are next to her and encourage her, she can relieve the pain a little. If she is also close to you to talk to you and encourage you, it helps you bear and relieve your pain a little*” (Pregnant woman with a history of C-section, hospital 7)*.*

In addition, when the companion is a person she trusts, the woman is reassured that everything that happens during the labor and birth will remain discreet and not gossiped about in the community. As one woman described:

“*We are reassured that the person will not say anything about you afterwards*” (Postpartum woman—C-section, hospital 6).

When the companion was a woman who had previously given birth herself, the support was perceived to be easier, because she had appropriate experience to understand the pain experienced by the woman giving birth and how to encourage her throughout labor and birth. For example, one companion described:

“*The advantage is that as they are between women, she really understands the pain and it is easier to assist her morally and really encourage her*” (Potential companion 4 of pregnant woman, hospital 6).

The companions could also help with practical support, such as by bringing to the women in labor the material that they need, including diapers and loincloths for wrapping the baby, or assist with the care of the baby and mother after birth:

“*Because you know that if you have someone, for example me who came here last night, when she finished giving birth we were told to send loincloths. But if you don't have someone, it's not the health workers who are going to go out and take the loincloth to come in. But since your family is there, when they (health workers) tell you to send a cloth, we just give it to you, at least that way it's good*.” (Companion 2 of postpartum woman, hospital 6).

#### Benefits for health providers

Health providers described three benefits of labor companions for them as health providers: communication, acting as a witness, and to help with caring. First, the companionship helped to facilitate good communication between the woman and the providers. This was particularly important for women who were unable to communicate with the healthcare providers in French, as the companions could then help translate communication from French to the woman’s local language:

“*It allows the exchange to be easy, the communication, because whatever you say the woman is under the effect of the pain and then there is the language barrier that is there. Therefore, some companions who understand French easily manage to help us communicate with our patients*” (Specialized nurse, hospital 5).

The companion was also perceived as a witness or watcher for the woman and her family. He/she could follow what happened during labor and different care received or not by the woman. If necessary, the companion could raise a complaint or concern about her care, as they were present to witness the type of care she received:

“*And since the companion is there directly: he/she follows the labor he/she sees how the care is done. It is rare that he/she complains after that patient has not been taken care of*” (Gynecologist, administrator, hospital 1).

Companions also helped with caring tasks, such as bringing the woman food or medications. This was particularly important given staffing constraints which limited the ability for midwives to complete these tasks for all women during busy periods, as described by this midwife:

“*It is an advantage when you cannot, at all times, make sure that there is someone who can go and get the medication and that it is not you. For example, if you have a woman who is in labor, who is alone, you are obliged to do everything for her, you are obliged to bring her food, you are obliged to go and get the medication for her, you are obliged to do everything, you are obliged to do what you should not do, because you are not going to leave her*” (Midwife, responsible of hospitalization ward, hospital 5).

#### The advantages for the companion

Health workers expressed beliefs that providing support to women throughout labor and birth may also have positive impacts on the labor companion themselves, as they will be able to understand what is happening throughout labor and birth (“*the people who are already accompanying, they have the real information*”) and provides them with new experiences. Providing labor companionship would also improve their ability to support a woman in a future birth, “*if you accompany someone, you know what happens and soon you will know what to expect*”. (Specialized nurse, in charge of care supervision, hospital 6).

#### Companionship and potential risks

Two potential risks of companionship were identified by women: (1) the companion may gossip or share private details about what happened during the birth, and (2) the companion may not be someone preferred or accepted by the woman. Women were concerned that other community members may tease them if intimate and private details were shared with people in the community, as described by this participant:

“*But there are some, when they accompany the woman in labor, all they are looking for is to go and tell. It is to see her intimacy, to hear everything she says and then to tell people*” (Potential companion 2 of pregnant woman, hospital 7)

Moreover, if the labor companion was someone who the woman did not choose herself, this can inflict further suffering on the woman. Having a companion present who was chosen by someone else—such as a mother-in-law or husband—may be an additional unwanted burden for the woman.

“*If we bring someone that she doesn't want to be with her, it's a double suffering that we inflict on her, but beyond that the concern is that at times it's difficult to have the one you want next to you if she sticks to that person and the person isn't next to her, it seems that it can complicate things*” (Companion 3 of postpartum woman, hospital 5)

#### Current role of companions and expected role

Participants recognized that currently, all women receive psychological, financial, or material support from a member of their family. However, typically the family member is not allowed to be continuously present throughout labor and birth and will wait outside the hospital while the baby is born. The current role of the companion is to act as an intermediary between the woman and the health workers.

“*That is to say, she needs to be assisted at all times when she has a concern and the follow-up is to follow her at all times so that she can have access to any health worker in a health center when she needs one*” (Potential companion 4, hospital 6).

All the health workers believed that the support from a labor companion should extend beyond the waiting room to stay with her during labor and childbirth, encourage and comfort her during pain.

### Enablers and barriers to implementation of WHO recommendations

#### Enabling factors

The existing model of companionship with the limitations on when the companion can be present (e.g. not during the birth, and intermittently throughout labor) is likely to help for smooth implementation of the WHO companionship model. Most of the women positively valued labor companionship as defined by WHO. Similarly, the health providers appreciated the idea of implementation of a more formal model of labor companionship, because this should allow clear characterization of the role of the companion. Health providers suggested that companions should be trained on infection prevention, hygiene, and the needs for woman’s well-being to fulfill their role.

The health policy environment in Burkina Faso could also act as an enabler. National guidelines already recommend that pregnant woman identifies a companion for birth and in case of emergency. It also states that during immediate postpartum, the health care provider must explain signs of danger to woman and her companion in order to call them if needed. These are acknowledgements of companionship that contribute to a policy-enabling environment in Burkina Faso. However, a limitation of the current policy is that the role of companion during labor and birth is not clearly defined.

#### Barriers

There are some barriers to the full implementation of the WHO companionship model: (1) limited space in the labor and delivery wards, (2) no private rooms for women, (3) family choosing a potentially undesirable companion for the woman, and (4) hospital rules prohibiting the presence of companion in the delivery room. In most of the study hospitals, access to the delivery room was not allowed to companions, because of limitations in the hospital infrastructures to preserve the privacy of women, as testified by a gynecologist:

“*No, first of all, the setting does not allow it, once the woman is in the delivery room, we cannot allow someone to be next to her because she is not alone, there are other women next to her*” (Gynecologist, hospital 7).

Currently, women did not typically choose their own companion. Rather, family—and particularly in-laws—choose the companion for the woman, which has the potential to limit her autonomy and choice. As one woman explained:

“*But here most of the time during the labor, you don't even choose, it's family that chooses a person for you to come and stay. You don't even know who she is. It's the day you leave for the maternity when you know that someone has been chosen to accompany you. You are there, you don't have a choice, it's the person that has been chosen for you who is there*” (Multiparous pregnant woman with a history of C-section, hospital 6).

For those who had the opportunity to choose, some criteria such as availability had to be taken into account as illustrated by this woman:

“*I had made a choice with a woman…but her work doesn't allow her to be able to accompany me to the maternity hospital for two or three days. So, I saw that if I said right away that she should go and ask her superiors for permission to come and sit with me, I was particularly disturbed. So, this option, I wanted her to assist me but I left it at once, because she told me that she had to go and ask. I told her no, it's okay, I changed my companion*” (Woman in postpartum, after C-section, hospital 7).

An additional factor that may affect implementation is the gender of the companion. Most companions were female, because labor and birth were considered as woman issues. Due to the lack of privacy in labor and delivery room, men were not allowed as stated by this health provider:

“*It’s true that the premises are not always very well equipped to receive a male companion, so most of the time, this may be the reason why the companions are female*” (Gynecologist, hospital 1).

More work may be needed ahead of implementation to explore potential options for men to attend, if they are the preferred companions for women.

## Discussion

We explored the perceptions and experiences of labor companionship in Burkina Faso among maternity service users and providers, and assessed the health facility readiness for implementation. Our study contributes important insights into improving the provision of labor companionship for women in Burkina Faso. The findings showed that the participants were unaware of the labor companionship model as defined in WHO recommendations. In addition, enabling factors and barriers that hinder implementation of WHO companionship model were identified. Some of these barriers have been well documented in both existing literature [19, 20] and in our study, such as limited space in labor and delivery wards, no private rooms or privacy measures (e.g., curtains) for women during labor, and hospital rules preventing companionship. However, an important consideration in Burkina Faso, and likely in other West African settings, includes that a woman may not be able to express her autonomy and choice about who supports her during this intimate time. In particular, there are important safety, confidentiality, and ethical concerns if her family or in-laws choose a companion for her who she does not desire. Moving forward to implementation, it will be critical to ensure that a woman’s preference about who supports her are respected, and more work is needed to understand how this can be navigated safely in these hospitals and other settings with similar social contexts.

Our findings showed that in Burkina Faso, labor companionship is socially accepted and is believed to have benefits for women, health providers, and companions. All participants were aware of labor companionship. Historically, women who gave birth in Burkina Faso were supported by experienced women—usually traditional birth attendants and their mothers-in-law. But since the 1970s, childbirth in Burkina Faso has progressively moved from homes and communities to health facilities. Even if women gives birth with assistance of health workers, their companions are still an important part of their experience [10]. All participants in our study acknowledged the existing companionship in early labor, which was currently limited to the waiting room and the post-natal period. However, none of the participants were aware about the WHO companionship model, which prioritizes the woman’s choice of who the companion is, and that they can be present continuously throughout labor and birth.

The different groups of participants (women, companions and health providers) unanimously recognized the benefits of companionship, particularly around the psychological support for women. They described that the presence of a companion gave confidence to the woman and encourages her to better bear pain for a successful outcome of the birth. Other studies have highlighted similar benefits [21, 22]. In addition, companions were helpful for health providers even if they are not allowed in labor and delivery rooms. Companionship established communication between the woman and the providers, but it also relieves the health providers from many non-medical tasks. Moreover, Lewis and Calves described how having a companion was like having a guardian against mistreatment from health workers in Burkina Faso [10]. On one hand, the absence of companion in the labor and delivery room reduces the pressure on women in terms of their pain control (she could cry and shout how she wants). On the other hand, this absence may contribute to experiences of mistreatment [10, 23]. Our study showed that women valued labor companionship in part due to the companion’s ability to act as a witness for poor treatment by healthcare providers. For these reasons, a companion during labor and birth may help women have a positive birth experience.

A qualitative study in Mali and Benin indicated increased use of C-section due to women’s and staff suffering and under-resourced facilities [24]. Encouraging companionship during labor may improve the quality of intrapartum care and outcomes for women and newborns, including decreasing C-section rates, by increasing emotional and informational support to women during labor, providing practical support and advocacy to the obstetrical team in the delivery room, and reducing pain and poor birth experiences [4]. However, the psychological benefits of companionship may be limited if the companion is not chosen by the woman, as revealed by some of our study participants.

The benefits of companionship perceived by women, health providers and companions were enabling factors for WHO companionship model in Burkina Faso. However, some critical challenges to implementing the WHO model of companionship exist that would need to be addressed ahead of implementation. Women raised issues related to mistrust in their companion and fears of companions gossiping about their birth experience, especially when it is not a companion of their choice [10]. Most companions were currently chosen by the woman’s family, especially in-laws which is likely a reflection of the historical roots of childbirth in Burkina Faso. In this country, traditionally, the mother-in-law takes care of her daughter-in-law during childbirth [10]. In addition, in Burkina Faso and elsewhere in Africa [25, 26], it is a tradition to bury the placenta as soon as it is expelled, in order to prevent the abduction of the placenta by spirits or witches [27]. For example, in Mossi culture (the majority ethnic group in Burkina Faso), the aunt of the woman’s husband or partner is responsible for the placenta, whereas in Yoruba culture, men are responsible for handling and disposal of the placenta [25]. This traditional practice around placental management may explain why in our study, a woman may be considered as a more appropriate and acceptable companion than a man. This is an important factor that may influence how labor companionship is implemented in Burkina Faso, as these cultural preferences and norms may simultaneously act as a barrier to the WHO model of companionship (only women can care for the placenta after birth, so only a woman can be a companion), and as a facilitator to companionship (justification for why the presence of a companion is important). Further exploration is needed to understand how traditional practices around the placenta may or may not be integrated into the labor companion models for Burkina Faso and any additional resources or support needed to maintain these traditional practices.

Finally, to fully implement the WHO labor companionship model in Burkina Faso, there are some important considerations that the QUALI-DEC project and other initiatives should address. First, in some cases, a female companion may be preferred to support cultural practices related to the placenta, or if there is a male companion, a female friend or family member may also need to be nearby to assist with the placenta. Second, labor and delivery rooms need to be furnished with curtains to allow privacy for women where it is possible. Finally, companions must be trained on their role and expectations from health care providers.

### Implications for policy and practice

National guidelines in Burkina Faso already recommend companionship for women; however, more work is needed to implementation of the WHO labor companionship model. At the national level, policymakers need to sensitize the population and promote companion of choice. The sensitization should provide clear message on the role of companion and the importance of the woman’s choice. Guidelines need to be revised to allow two companions per woman that will be socially acceptable. Finally, in response to recommendations for companionship, the design of future maternity wards should account for privacy and intimacy of labor and delivery rooms through the use of curtains, partitions, or private rooms. At the hospital level, the management team should provide equipment such as curtains for labor wards where structural changes are difficult to implement. Appropriate information to women’s companions on their expected roles upon their arrival needs to be available and actively distributed. Finally, hospitals must adapt their policies to allow the continuous presence of companions during labor and birth, including how to ensure companionship during caesarean section.

### Strengths and limitations

Our study had both limitations and strengths. Key strengths include the triangulation of qualitative research and facility readiness assessment to provide a holistic understanding of hospital environment and context, as well as peoples’ perceptions and experiences in providing and receiving care. Our sample was limited to hospitals that provided C-section and vaginal births and had high rates of C-section. While many vaginal births in Burkina Faso occur at the primary health facility level, barriers and facilitators of companionship at that level may be different in these settings, and more work is needed to understand which factors may be transferrable to lower-level health facilities. Our participant sample (women and companions) was limited to those who gave birth at the hospital; women who gave birth at home were not included. Even if it was relevant to include them, currently in Burkina Faso there are few women who still give birth at home.

## Conclusion

Our qualitative study analyzed women’s and providers’ perceptions of labor companionship and showed that all stakeholders are aware of the advantages of companionship, but more work is needed to adapt the WHO labor companionship model for culturally appropriate implementation in Burkina Faso. Women have high expectations of this type of support, as its implementation could encourage them to do their best to ensure that their labor and birth goes smoothly.

## Data Availability

The datasets used and/or analyzed during the current study are available from the corresponding author on reasonable request.
